# Profiling of testis-specific long noncoding RNAs in mice

**DOI:** 10.1186/s12864-018-4931-3

**Published:** 2018-07-16

**Authors:** Seong Hyeon Hong, Jun Tae Kwon, Jihye Kim, Juri Jeong, Jaehwan Kim, Seonhee Lee, Chunghee Cho

**Affiliations:** School of Life Sciences, Gwangju Institute of Science and Technology, Gwangju, 61005 Korea

**Keywords:** *cis*-acting, Long noncoding RNA, Spermatogenesis, Testis, Testis-specific lncRNAs

## Abstract

**Background:**

Spermatogenesis, which is the complex and highly regulated process of producing haploid spermatozoa, involves testis-specific transcripts. Recent studies have discovered that long noncoding RNAs (lncRNAs) are novel regulatory molecules that play important roles in various biological processes. However, there has been no report on the comprehensive identification of testis-specific lncRNAs in mice.

**Results:**

We performed microarray analysis of transcripts from mouse brain, heart, kidney, liver and testis. We found that testis harbored the highest proportion of tissue-specific lncRNAs (11%; 1607 of 14,256). Testis also harbored the largest number of tissue-specific mRNAs among the examined tissues, but the proportion was lower than that of lncRNAs (7%; 1090 of 16,587). We categorized the testis-specific lncRNAs and found that a large portion corresponded to long intergenic ncRNAs (lincRNAs). Genomic analysis identified 250 protein-coding genes located near (≤ 10 kb) 194 of the loci encoding testis-specific lincRNAs. Gene ontology (GO) analysis showed that these protein-coding genes were enriched for transcriptional regulation-related terms. Analysis of male germ cell-related cell lines (F9, GC-1 and GC-2) revealed that some of the testis-specific lncRNAs were expressed in each of these cell lines. Finally, we arbitrarily selected 26 testis-specific lncRNAs and performed in vitro expression analysis. Our results revealed that all of them were expressed exclusively in the testis, and 23 of the 26 showed germ cell-specific expression.

**Conclusion:**

This study provides a catalog of testis-specific lncRNAs and a basis for future investigation of the lncRNAs involved in spermatogenesis and testicular functions.

**Electronic supplementary material:**

The online version of this article (10.1186/s12864-018-4931-3) contains supplementary material, which is available to authorized users.

## Background

Spermatogenesis is the complex and tightly regulated differentiation process through which haploid spermatozoa are produced in the seminiferous tubules of the testis. This process can be divided into three successive phases: the mitotic division of spermatogonia, the meiosis of spermatocytes and the morphological change of spermatids during spermiogenesis [1]. It is expected that a highly organized intrinsic genetic network is responsible for controlling spermatogenesis in the testis, and that the elucidation of the underlying molecular mechanism will help us further understand male germ cell development. The previous studies have largely focused on identifying and characterizing protein-coding genes [2] and small noncoding RNAs, such as microRNAs (miRNAs) [3] and piwi-interacting RNAs (piRNAs) [4], in the mouse testis. However, a recent transcriptomic analysis revealed that there is pervasive transcription of long noncoding RNAs (lncRNAs) in the mouse testis [5].

LncRNAs are arbitrarily defined as noncoding RNAs longer than 200 nucleotides. Compared with protein-coding transcripts, they tend to be shorter and have lower expression levels, fewer exons, less sequence conservation and more tissue and cell type-specific expression patterns [6–11]. Studies have shown that lncRNAs are novel regulatory molecules involved in processes of genetic regulation, including transcription [12, 13], epigenetic modification [14, 15], alternative splicing of pre-mRNAs [16, 17] and mRNA stabilization or decoy functions [18, 19]. lncRNAs have also been shown to play important roles in other biological processes, including cell differentiation [19–21] and tissue development [21, 22].

Recent studies have identified lncRNAs in the mouse testis and examined their biological roles during male germ cell development [23–27]. However, although numerous studies have investigated the protein-coding genes that show tissue-specific expression and play important roles during spermatogenesis, no previous study has comprehensively identified and characterized the mouse testis-specific lncRNAs.

Here, we used microarray analysis to profile the previously annotated lncRNAs and mRNAs expressed in various mouse tissues, including testis. By comparing the lncRNA and mRNA expression profiles among the tissues, we identified lncRNAs and mRNAs that showed differential expression in each tissue. We found that testis has the largest number of tissue-specific lncRNAs. We also profiled lncRNA and mRNA expression in three mouse male germ cell-related cell lines (F9, GC-1 and GC-2). To the best of our knowledge, this is the first study to identify mouse testis-specific lncRNAs and analyze their characteristics. Our data provide a valuable resource for future investigation of the lncRNAs that are involved in male germ cell development and testicular functions.

## Results

### Identification of lncRNAs and mRNAs in mouse tissues

**Fig. 1:**
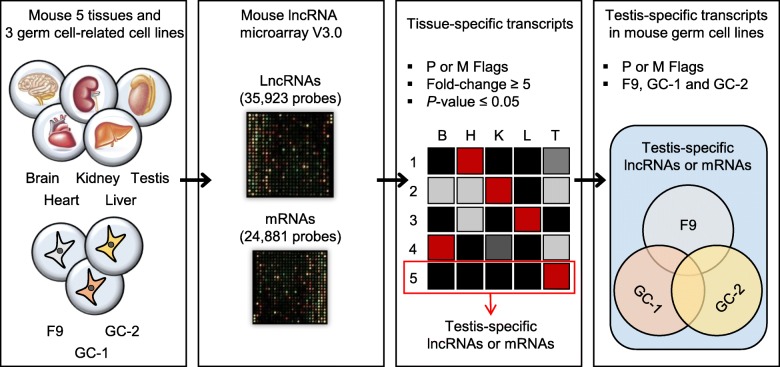
Schematic diagram of the expression profiles of lncRNAs and mRNAs in mouse major tissues and germ cell-related cell lines

To identify lncRNAs and mRNAs expressed in major tissues (brain, heart, kidney, liver and testis) in ICR mice and germ cell-related cell lines (F9, GC-1 and GC-2) in mice, we performed a microarray analysis using the Arraystar mouse lncRNA microarray V3.0, which contains probes for 35,923 lncRNAs and 24,881 mRNAs (Fig. 1). The expression profiles obtained from this analysis are summarized in Table 1 and Additional file 1.

**Table 1:** Summary of the microarray results obtained in five mouse tissues and three mouse cell lines

	lncRNAs	mRNAs	lncRNAs	mRNAs
Tissues	Total expressed		Specifically expressed	
Brain	12,832	15,579	595	553
Heart	10,086	13,354	104	89
Kidney	9915	13,319	102	106
Liver	6290	9431	48	76
Testis	14,256	16,487	1607	1090
Germ cell-related cell lines	Total expressed		Testis-specific	
F9	9065	12,512	128	187
GC-1	10,571	13,492	143	167
GC-2	10,652	13,443	126	175

**Fig. 2:**
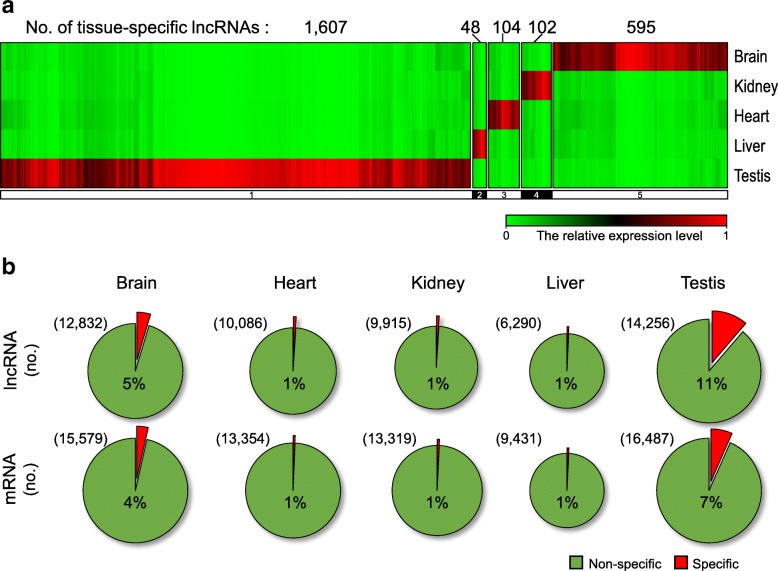
Characteristics of the identified tissue-specific lncRNAs and mRNAs. **a** Heat map showing the relative expression levels of the lncRNAs that exhibited differential expression in five major mouse tissues. Red and green colors indicate higher and lower expression levels, respectively. **b** The percentage of tissue-specific lncRNAs and mRNAs in each mouse tissue

The maximal numbers of both lncRNAs (14,256) and mRNAs (16,587) were observed in testis compared to the other tissues, suggesting that testis is characterized by a high level of transcriptomic diversity and complexity. To perform tissue-specific expression profiling, we compared the expression levels of transcripts among the five tissues across three microarray experiments and classified the transcripts as present (P), marginal (M) or absent (A) of which expression level was lower than the background level. We regarded a transcript as a tissue-specific lncRNA or mRNA with the expression showing a fold-change ≥5 and *P*-value ≤0.05 for a tissue relative to the others and with three independent P or M only in the tissue. We found that testis exhibited the largest numbers of tissue-specific lncRNAs (1607) and mRNAs (1090) among the analyzed tissues (Table 1, Fig. 2a and Additional file 2). The testis-specific lncRNAs were more abundant than the testis-specific mRNAs, even though there were fewer total lncRNAs than mRNAs in this tissue (Fig. 2b). We speculated that these newly identified testis-specific lncRNAs could be involved in regulating testicular gene expression and/or functions.

**Fig. 3:**
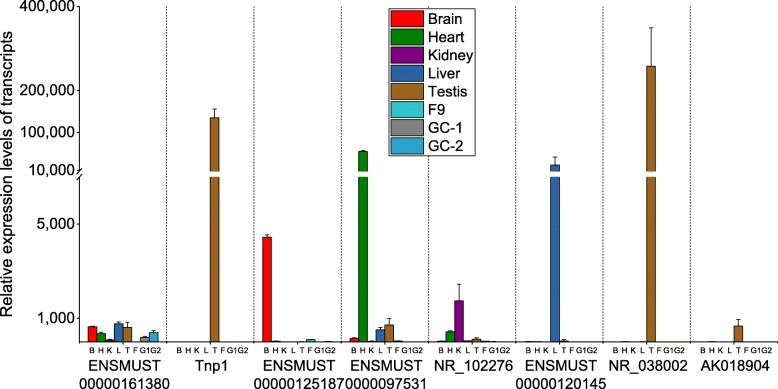
qRT-PCR-based validation of six arbitrarily selected tissue-specific lncRNAs. Data show the mean ± SEM of triplicates for each sample

To validate our microarray-based selection of tissue-specific lncRNAs, we performed quantitative real-time PCR (qRT-PCR) on several arbitrarily selected tissue-specific lncRNAs (Fig. 3). As controls, we used ENSMUST00000161380, an lncRNA that is predicted to be ubiquitously expressed and the transition protein 1 (*Tnp1*) gene, which encodes a testis-specific mRNA [28]. The tissue-specific lncRNAs subjected to qRT-PCR analysis were ENSMUT00000125184 (brain), ENSMUST00000125187 (heart), NR_102276 (kidney), ENSMUST00000120145 (liver), NR_038002 (testis) and AK018904 (testis). Our qRT-PCR analysis demonstrated that these lncRNAs were specifically or predominantly expressed in the corresponding tissues (Fig. 3), confirming our microarray data.

### Chromosomal distribution of testicular lncRNAs

**Fig. 4:**
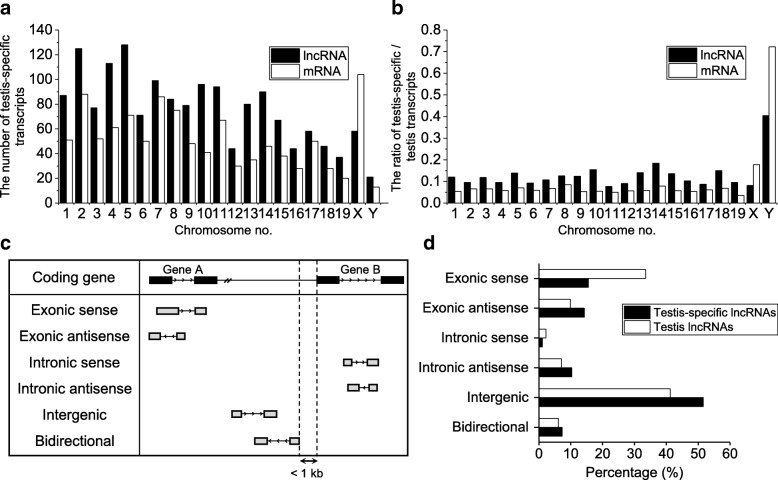
Chromosomal distribution and classification of testicular lncRNAs. **a** The number of testis-specific transcripts (lncRNAs and mRNAs found to be specifically expressed in testis through the microarray analysis) on each chromosome. **b** The ratio of testis-specific transcripts to testicular transcripts on each chromosome. The ratio was obtained by dividing the number of testis-specific transcripts by the number of total testicular transcripts. **c** lncRNAs were classified into six subtypes (exonic sense, exonic antisense, intronic sense, intronic antisense, intergenic, and bidirectional) based on the positional and directional relationship between their genomic regions and those of nearby mRNAs. **d** The percentages of the six subtypes among the testicular lncRNAs

To characterize the genomic nature of the identified testicular lncRNAs and mRNAs, we examined the chromosomal localization of the loci that expressed these transcripts. Our analysis revealed that they were widely distributed throughout the mouse chromosomes. Chromosome 5 and the X chromosome contain the highest numbers of loci for testis-specific lncRNAs and mRNAs, respectively (Fig. 4a and Additional file 3). To further examine whether one or more chromosomes were enriched with testis-specific lncRNAs and mRNAs, we compared the ratio of testis-specific transcripts to total testicular transcripts among chromosomes (Fig. 4b). The ratio was similar among the chromosomes, except for the sex chromosomes: the ratios of testis-specific lncRNAs and mRNAs were both remarkably high for the Y chromosome, even though relatively few loci on this chromosome expressed testis-specific transcripts (21 testis-specific lncRNAs and 13 testis-specific mRNAs). Based on this finding, we speculate that the expressions of testis-specific lncRNAs and mRNAs may be related on the Y chromosome, perhaps through *cis*-regulation between the two groups of testis-specific transcripts.

### Classification of testicular lncRNAs

We categorized all of the identified lncRNAs based on the position and direction of their genomic regions relative to nearby mRNAs. The lncRNAs were classified into six subtypes: exonic sense, exonic antisense, intronic sense, intronic antisense, intergenic and bidirectional (Fig. 4c and Additional file 4). If an lncRNA overlapped with the exon or intron of a coding gene with the same or opposite direction, it was called exonic sense, exonic antisense, intronic sense or intronic antisense, respectively. A bidirectional lncRNA was defined as a transcript whose locus was less than 1 kb from an mRNA-encoding region, regardless of its direction. Finally, a genomic locus more than 1 kb from any mRNA-encoding genomic region was considered to be a long intergenic ncRNA (lincRNA). Using this classification scheme, we found that the majority of testis-specific (51.6%) and testicular (41.2%) lncRNAs were lincRNAs (Fig. 4d).

Because many lincRNAs are known to act in a *cis*-regulatory manner [29, 30], we investigated the potential *cis*-regulatory targets of 829 testis-specific lincRNAs by searching for protein-coding genes within 10 kb up- and downstream of their encoding loci. We found that 23.4% (194 of 829) of the lincRNAs co-localized with 250 protein-coding genes (Additional file 5). Interestingly, some of these genes are testis-specific or predominant genes (e.g., *Tnp1, Glt6d1, 1700018C11Rik, Speer4e, Spata31d1d, Tmco5, Spata17, 1700080E11Rik, Rcc1, Agbl3* and *1700057G04Rik)*, suggesting the expression regulation of these genes by the lincRNAs through transcriptional or post-transcriptional mechanisms. Gene Ontology (GO) [31] analysis of all of the identified nearby protein-coding genes was performed to explore their functions. We found that 40 diverse GO terms were significantly enriched (*P*-value ≤0.05), including regulation of transcription DNA-templated (GO:0006355), transcription DNA-templated (GO:0006351), positive regulation of transcription from RNA polymerase II promoter (GO:0045944), negative regulation of transcription from RNA polymerase II promoter (GO:0000122) and cell differentiation (GO:0030154) (Additional file 6). This suggests that the testis-specific lincRNAs could potentially modulate the expression of genes that encode proteins involved in transcriptional regulation.

### Conservation of mouse testis-specific lncRNAs in human

Although lncRNAs generally show poor sequence conservation among species [8], we utilized the BLASTN program [32] to examine the sequence conservation between human lncRNAs and the mouse testis-specific lncRNAs identified in this study. Starting from the 1607 mouse testis-specific lncRNAs, we obtained genomic sequence information for 1508 (93.8%) from diverse genome databases (e.g., UCSC genome browser, NCBI and Ensemble). When we used an *E*-value ≤10^− 5^ as a cut off, we found that only 79 (5.2%) of the mouse testis-specific lncRNAs were partially conserved in the human genome (Additional file 7). This indicates that testis-specific lncRNAs have low primary sequence conservation, and are thus likely to experience rapid evolution.

### Expression of testis-specific lncRNAs in germ cell lines

**Fig. 5:**
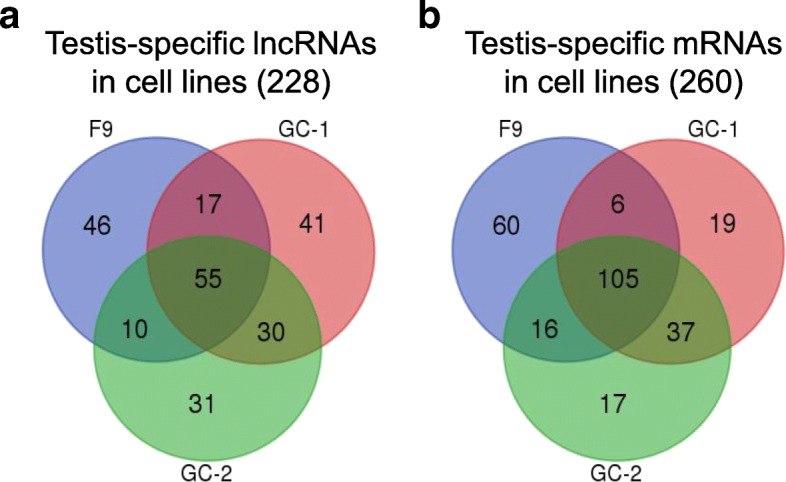
Venn diagram showing the number of testis-specific lncRNAs and mRNAs in three mouse germ cell-related cell lines. **a** Two hundred twenty eight of the testis-specific lncRNAs were identified across the three cell lines. **b** Two hundred sixty testis-specific mRNAs were identified across the three cell lines

To further characterize the testis-specific lncRNAs, we used microarray analysis to investigate their expression profiles in three relevant cell lines: F9, GC-1 and GC-2 (Fig. 1 and Additional file 8). F9 embryonal carcinoma cells are stem cells of testicular carcinoma, and have the ability to differentiate into three embryonic layers [33]. Although F9 cells are considered to be similar to embryonic cells, the former can transcribe germ cell-specific genes and possess the characteristics of male germ cells [34]. Immortalized GC-1 cells exhibit the characteristics of a stage between type B spermatogonia and primary spermatocytes [35], while GC-2 cells were derived from cells arrested at a premeiotic stage and exhibit the characteristics of spermatocytes [36]. Our analysis revealed that some of the testis-specific lncRNAs (14%; 228 of 1607) and mRNAs (23%; 260 of 1090) were detectable in one or more of the cell lines. When assessed individually, the various cell lines were found to express 128 (F9), 143 (GC-1) and 126 (GC-2) of the lncRNAs and 187 (F9), 167 (GC-1) and 175 (GC-2) of the mRNAs (Fig. 5 and Additional file 9). Thus, these cell lines should prove useful for the further characterization of certain testis-specific lncRNAs and mRNAs.

### In vitro expression of testis-specific lncRNAs

**Fig. 6:**
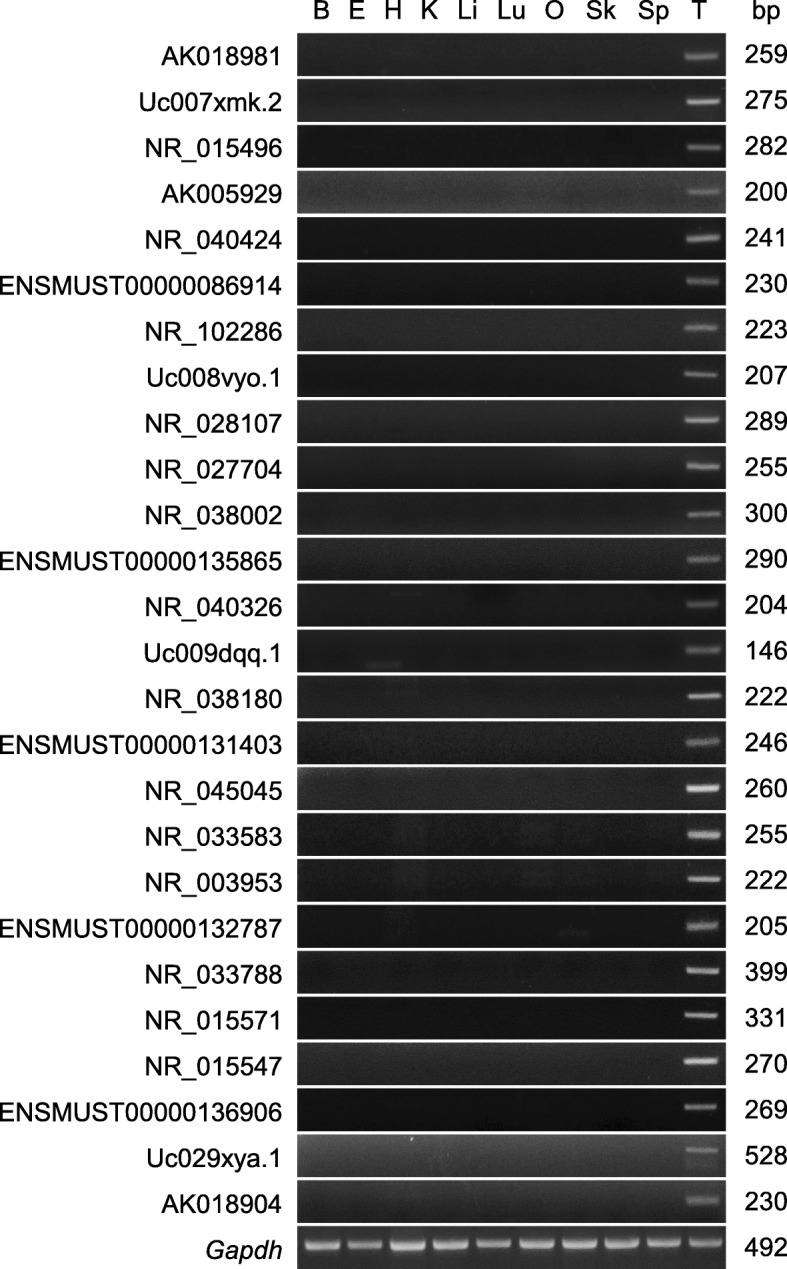
Tissue distribution of 26 arbitrarily selected novel testis-specific lncRNAs in 10 mouse tissues, as assessed by RT-PCR. The results were normalized with respect to the band intensity of glyceraldehyde-3-phosphate dehydrogenase (*Gapdh*). B, Brain; E, Epididymis; H, Heart; K, Kidney; Li, Liver; Lu, Lung; O, Ovary; Sk, Skeletal muscle; Sp, Spleen; and T, Testis

As we initially identified our tissue-specific transcripts using only five tissues in our microarray analysis, we assessed whether the selected lncRNAs were indeed testis-specific by selecting 26 of them predicted to have relatively abundant expression, and using reverse transcription-polymerase chain reaction (RT-PCR) to examine their distribution in 10 mouse tissues. Our results showed that all 26 of the testis-specific lncRNAs were transcribed only in the mouse testis (Fig. 6).

**Fig. 7:**
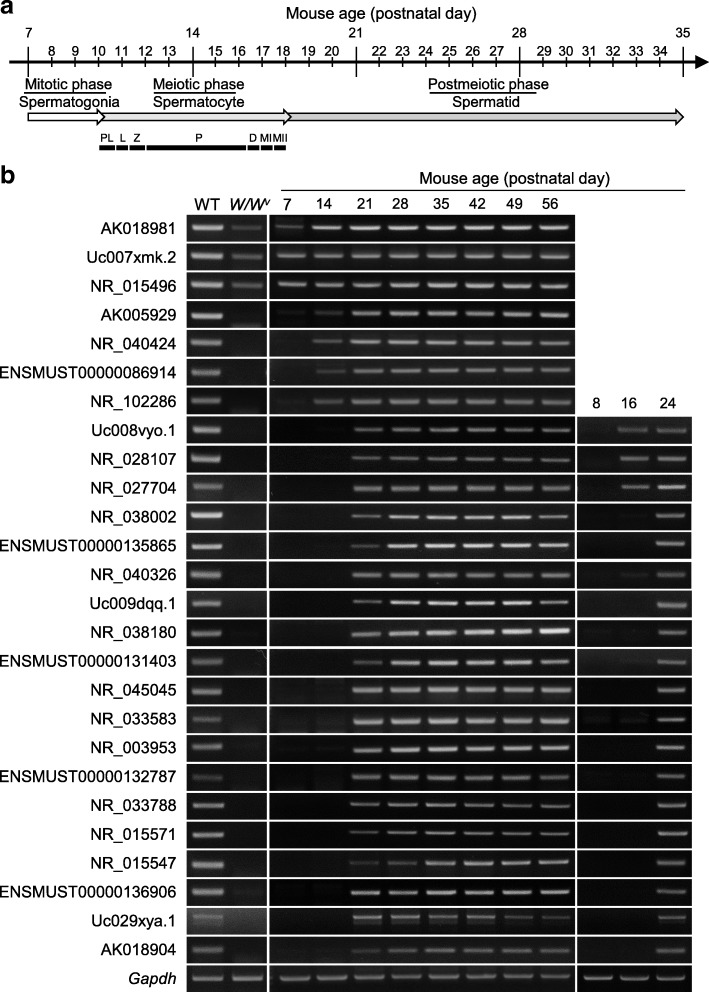
Developmental expression patterns of the 26 novel testis-specific lncRNAs during the first round of spermatogenesis. **a** Schematic diagram showing the first round of spermatogenesis, which is composed of the mitotic, meiotic and postmeiotic phases. The meiotic phase consists of preleptotene (PL), leptotene (L), zygotene (Z), pachytene (P), diplotene (D), meiotic division I (MI) and meiotic division II (MII). **b** Developmental expression patterns of testis-specific lncRNAs in germ cell-lacking testes (*W/W*^*v*^ mutant mice) and wild-type mouse testes were assessed by RT-PCR of samples taken at different postnatal days

Next, we examined the germ cell-specific expression patterns of these 26 testis-specific lncRNAs using the germ cell-less testis of *W/W*^*v*^ (c-kit) mutant mice [37]. This analysis showed that 23 of the testis-specific lncRNAs were absent from the testis of *W/W*^*v*^ mutant mice (Fig. 7b), suggesting that they are germ cell-specific lncRNAs. The other three testis-specific lncRNAs may also be expressed in somatic cells of the testis.

Finally, we examined the developmental expression of these 26 testis-specific lncRNAs in mouse testis at different postnatal days. Spermatogonial stem cells gradually proliferate and differentiate to produce spermatogonia, spermatocytes and round spermatids during the first round of spermatogenesis in the prepubertal mouse [38]. Meiosis begins in the mouse testis at postnatal day 10. At postnatal day 14, pachytene spermatocytes are highly enriched to about one-third of the total cells in the seminiferous tubules. Round spermatids appear in the seminiferous tubules at postnatal day 18 (Fig. 7a). If the testis-specific lncRNAs are expressed only in germ cells during the first round of spermatogenesis, they will first appear in the testis at a postnatal time that corresponds to a specific stage of spermatogenesis. Indeed, we found that four of the testis-specific lncRNAs were first expressed at postnatal day 14, suggesting that they are expressed in early-stage spermatocytes; three were first expressed at postnatal day 16, suggesting that they are expressed from pachytene spermatocytes; and 16 were first expressed at postnatal day 21, suggesting that they were expressed from round spermatids (Fig. 7b). These results suggest that most of the identified testis-specific lncRNAs are expressed specifically in spermatogenic cells, and their transcription appears to be regulated in a stage-dependent manner.

## Discussion

Recent studies have shown that lncRNAs play important roles in regulating testicular development and spermatogenesis [26, 27]. Testis lncRNAs have been identified in mammalian species, including mouse [23–25], rat [39], pig [40] and human [5]. In mice, several studies have investigated the expression profiles of testis lncRNAs during postnatal development [23], during specific developmental stages of spermatogenesis [24] and at critical time points during male germ cell development [25]. In order to understand the unique and complicated features of the transcriptome in testis, we need to identify lncRNAs that are differentially expressed in testis relative to other tissues [5]. However, no previous study had set out to identify lncRNAs that are specifically or predominantly expressed the testis of mouse. To address this gap, we herein used microarray analysis to identify tissue-specific lncRNA transcripts in mouse brain, heart, kidney, liver and testis. We identified 14,256 lncRNAs and 16,487 mRNAs that were significantly expressed in mouse testis. Of them, 1607 lncRNAs and 1090 mRNAs were testis-specific. Among the studied tissues, testis had the most tissue-specific lncRNAs (11%) and mRNAs (7%). Notably, lncRNAs exhibited more testis specificity than mRNAs, suggesting that lncRNAs may be involved in male germ cell development.

We observed that the testis-specific lncRNAs and mRNAs were enriched on the Y chromosome relative to the other mouse chromosomes. Genes of the mammalian Y chromosome are known to play important roles in sex determination (e.g., *Sry*) [41] and spermatogenesis (e.g., *Eif2s3y*) [42], suggesting that the testis-specific lncRNAs of the Y chromosome deserve further investigation. The functions of lncRNAs cannot be predicted due to their poor sequence conservation and our lack of knowledge regarding relevant functional motifs and domains in protein-coding genes. However, a number of studies have inferred the putative functions of lncRNAs by examining their genomic relationships with protein-coding genes, and some lncRNAs have been shown to regulate the expression of their overlapping or neighboring protein-coding genes [43]. We herein identified several subtypes of lncRNAs, and found that lincRNAs constituted the largest proportion of both testis and testis-specific lncRNAs [10]. We hypothesized that the testis-specific lincRNAs could act through a *cis*-regulatory mechanism, and thus identified and examined 250 protein-coding genes within the 10-kb regions up- and downstream of 194 testis-specific lincRNAs. Our GO analyses revealed that these genes were mainly involved in regulating transcription. It seems possible that the testis-specific lincRNAs could contribute to spermatogenesis through such function. Interestingly, some of the identified *cis*-acting target protein-coding genes are known to be involved in spermatogenesis (e.g., *Tnp1* and *Spata17*), raising the possibility that the testis-specific lincRNAs could play direct regulatory roles in male germ cell development. Alternatively, they may act in *trans* by targeting protein-coding genes located on the other mouse chromosomes. To answer whether testis-specific lincRNAs indeed regulates the expression of these protein-coding genes requires further investigation.

Although it was previously reported that lncRNAs have a very low sequence conservation among species [8], we used BLAST analysis to investigate the sequence conservation of the mouse testis-specific lncRNAs with human lncRNAs. However, relatively few mouse testis-specific lncRNAs (5.2%) were found to be conserved in the human lncRNAs. We speculate that the testis-specific lncRNAs may not require sequence conservation to maintain their functionality. Therefore, in the future it will be important to examine the short conserved sequences and/or secondary structures of testis-specific lncRNAs.

We used microarray analysis to examine the expression profiles of the selected lncRNAs and mRNAs in the cell lines, F9, GC-1 and GC-2 [], which exhibit germ cell characteristics. A total of 128, 143 and 126 testis-specific lncRNAs and 187, 167 and 175 testis-specific mRNAs were found to be expressed in the F9, GC-1 and GC-2 cell lines, respectively. Fifty five lncRNAs and 105 mRNAs were expressed in an overlapping manner in all of the cell lines. It is possible that these transcripts are expressed during the whole period of spermatogenic stages from which the cell lines were derived. It should be noted that these cell lines do not possess all the characteristics and phenotypes of male germ cells, such as meiosis and spermiogenesis, suggesting weak and/or no expression of some of the testis- or germ cell-specific transcripts in the cell lines. Nonetheless, these cell lines could prove useful for studying the expression and functions of certain testis-specific lncRNAs.

We arbitrarily selected 26 of the novel testis-specific lncRNAs and examined their tissue distributions in 10 mouse tissues. All of the examined lncRNAs were, indeed, specifically expressed in the mouse testis. Additionally, both developmental expression analysis during the first round of spermatogenesis and expression analysis in germ cell-less mutant mice confirmed the germ cell-specificity of these lncRNAs. Interestingly, 16 of the 26 selected lncRNAs were expressed between 16 and 21 days after birth in mouse, suggesting that these lncRNAs undergo a transcriptional change between the late spermatocyte and haploid round spermatid stages. During this period, epigenetic regulation (e.g., DNA methylation and histone modification) occurs actively in testis [44]. As lncRNAs have been implicated in regulating protein-coding genes at the epigenetic level [45], we speculate that these germ-specific lncRNAs may be involved in epigenetic regulation during spermatogenesis.

## Conclusions

We herein used microarray analysis to identify and characterize lncRNAs in mice, and then focused on comprehensively cataloging the lncRNAs specifically found in the testis, which showed the highest proportion of tissue-specific expression. Our findings provide a basis for further investigating the functions of testis-specific lncRNAs, which should improve our understanding of the lncRNA-mediated regulatory mechanisms that may be associated with mouse germ cell development.

## Methods

### Animals and tissue preparation

All animal experiments were performed in accordance with Korean Food and Drug Administration (KFDA) guidelines. Protocols were reviewed and approved by the Institutional Animal Care and Use Committees (IACUC) of Gwangju Institute of Science and Technology (GIST) (permit number: GIST-2017-013). We used 8-week-old ICR male mice (Damul Science, Daejeon, Korea) for our microarray analysis. Mouse brain, heart, kidney, liver and testis tissues were obtained and immediately frozen in liquid N_2._ Three biological replicates were performed in all cases, and samples were stored at − 80 °C until use.

### Cell culture

F9 (CRL-1720), GC-1 (CRL-2053) and GC-2 (CRL-2196) cells of mouse origin were obtained from the American Type Culture Collection (ATCC, Manassas, VA, USA) and maintained at 37 °C and 5% CO_2_ in Dulbecco’s modified Eagle’s medium (DMEM) (Life Technologies, Carlsbad, CA, USA) supplemented with 10% fetal bovine serum (HyClone, Logan, UT, USA). The culture dishes for the F9 cells were pre-coated with 0.1% gelatin. Because retinoic acid (RA) is known to induce F9 cell differentiation [46], it was omitted from the medium. The culture media were changed every 1–2 days, and the cells were sub-cultured every 3–4 days. At passages 3 or 4, cultured cells were collected for RNA extraction.

### RNA extraction, quality control and labeling

Total RNA from five major mouse tissues and three mouse germ cell-related cell lines was isolated with an RNeasy Plus Mini kit (Qiagen, Valencia, CA, USA) according to the manufacturer’s instructions. Total RNA was quantified and checked for quality using an ND-1000 spectrophotometer (Thermo Fisher Scientific, Waltham, MA, USA). RNA degradation and DNA contamination were examined by 1% denaturing agarose gel electrophoresis. RNA samples with RNA Integrity Number (RIN) scores > 8 were used for microarray analysis. Each 1 μg of total RNA was reverse-transcribed into cDNA using a MMLV-RT kit (Life Technologies). The cDNA was transcribed and labeled with Cyanine 3-CTP (Cy3) using a Quick-Amp labeling kit (Agilent, Santa Clara, CA, USA) according to the manufacturer’s protocol. The labeled cRNAs were quantified using an ND-1000 spectrophotometer.

### Mouse lncRNA microarray analysis

Total cRNA was hybridized to a Mouse LncRNAs Microarray (8 × 60 K; Arraystar) using a hybridization oven (Agilent). The hybridized microarray chip was washed according to the manufacturer’s protocol, and the hybridized images were scanned using a DNA microarray scanner and quantified with the Feature Extraction Software (both from Agilent). Data normalization and selection of fold-changed transcripts were performed using GeneSpring GX 7.3 (Agilent). Total lncRNAs and mRNAs were detected in three independent sets of the samples in each tissue; those above background were flagged as present or marginal (P or M, respectively). For each tissue, specifically expressed lncRNAs and mRNAs were chosen using the following criteria: 1) in three independent samples, they were flagged as P or M in a single given tissue but absent (A) in the other tissues; 2) fold-change ≥5; and 3) *P*-value ≤0.05. The raw and processed microarray data have been deposited in the Gene Expression Omnibus (GEO) database at the National Center for Biotechnology Information (NCBI), under GEO accession number GSE105024.

### Validation of tissue-specific lncRNAs by qRT-PCR

Total RNA was extracted from five mouse tissues and three germ cell-related cell lines, and reverse-transcribed using RNeasy Plus Mini and Omniscript RT kits (Qiagen). qRT-PCR was carried out using the TOPreal™ qPCR 2X premix (Enzynomics, Daejeon, Korea). The reaction volume contained 10 μl of TOPreal™ qPCR 2X PreMix, 1 μl of 10 μM forward and reverse primers, 50–100 ng of template cDNA and dH_2_O to a final volume of 20 μl. The reactions were performed on a StepOnePlus Real-Time PCR System (Thermo Fisher Scientific) as follows: 95 °C for 12 min 30 s, followed by 40 cycles of 95 °C for 10 s, 60 °C for 15 s and 72 °C for 23 s. The melting curve analysis was performed from 65 °C to 95 °C with increments of 0.3 °C. The primer sets used to amplify the selected lncRNAs and control genes are listed in Additional file 10. Amplifications were performed in triplicate for each sample. Relative gene expression levels were evaluated using the 2^-ΔΔCt^ method [47], and normalized with respect to the level of the endogenous *Gapdh* mRNA*.*

### Searching for nearby protein-coding genes and gene ontology analysis

As several lincRNAs act as through a *cis*-regulatory mechanism [29, 30], we searched for protein-coding genes located in the genomic regions 10 kb up- and downstream of the testis-specific lincRNAs. We then predicted their functional roles by GO analysis using the DAVID software [48]. GO terms with *P*-value ≤0.05 were considered significantly enriched.

### RT-PCR analysis of tissue distribution and developmental expression patterns

To determine the tissue distribution patterns of 26 testis-specific lncRNAs arbitrarily selected from our microarray analysis, we performed RT-PCR using cDNA from 10 mouse tissues (brain, epididymis, heart, kidney, liver, lung, ovary, skeletal muscle and testis). We also used cDNA from germ cell-lacking testes of *W/W*^*v*^ mutant mice to investigate whether these lncRNAs were expressed in somatic cells of testis [37]. To investigate the developmental expression patterns of the selected lncRNAs during the first round of spermatogenesis, total RNA from testes of prepubertal and adult mice (7d, 8d, 14d, 16d, 21d, 24d, 28d, 35d, 42d, 49d and 56d) was subjected to RT-PCR. Total RNA was extracted using the TRIzol reagent (Invitrogen, Carlsbad, CA, USA) according to the manufacturer’s protocol, and cDNA was synthesized using random hexamers, oligo(dT) primers and Omniscript reverse transcriptase (Qiagen). lncRNA-specific primers were designed using Primer-BLAST and are listed in Additional file 11. Amplification was performed for 35–40 cycles of 95 °C for 30 s, 50 °C or 55 °C for 30 s and 72 °C for 30 s. Glyceraldehyde-3-phosphate dehydrogenase (*Gapdh*) were used as a loading control.

### Statistical analyses

All experiments were performed in triplicate for each sample. Statistical analysis was performed using the Student’s *t*-test, and *P* ≤ 0.05 was taken as indicating a significant difference. Data are presented as the mean ± standard error of the mean (mean ± SEM).

## Additional files

Additional file 1: **Table S1.** LncRNAs and mRNAs in the mouse brain, heart, kidney, liver and testis. (XLSX 18613 kb)

Additional file 2: **Table S2.** Tissue-specific lncRNAs and mRNAs in the mouse brain, heart, kidney, liver and testis. (XLSX 4020 kb)

Additional file 3: **Table S3.** The numbers and ratios of testicular lncRNAs and mRNAs on each mouse chromosome. (XLSX 11 kb)

Additional file 4: **Table S4.** The numbers and ratios of six types of testicular lncRNA. (XLSX 10 kb)

Additional file 5: **Table S5.** Nearby protein-coding genes located within genomic regions 10 kb up- and downstream of the testis-specific lincRNAs. (XLSX 24 kb)

Additional file 6: **Table S6.** GO terms of the nearby protein-coding genes. (XLSX 19 kb)

Additional file 7: **Table S7.** Mouse testis-specific lncRNAs that show conservation in human. (XLSX 26 kb)

Additional file 8: **Table S8.** LncRNAs and mRNAs in three mouse germ cell-related cell lines (F9, GC-1 and GC-2). (XLSX 16402 kb)

Additional file 9: **Table S9.** Testis-specific lncRNAs and mRNAs in three mouse germ cell-related cell lines (F9, GC-1 and GC-2). (XLSX 712 kb)

Additional file 10: **Table S10.** Primers used for qRT-PCR. (XLSX 10 kb)

Additional file 11: **Table S11.** Primers used for RT-PCR. (XLSX 11 kb)

## Abbreviations

Agbl3: ATP/GTP binding protein-like 3
Eif2s3y: Eukaryotic translation initiation factor 2, subunit 3, structural gene Y-linked
Glt6d1: Glycosyltransferase 6 domain containing 1
GO: Gene ontology
lincRNA: Long intergenic ncRNA
lncRNA: Long noncoding RNA
miRNA: microRNAs
piRNA: Piwi-interacting RNA
qRT-PCR: Quantitative real-time PCR
Rcc1: Regulator of chromosome condensation 1
Spata17: Spermatogenesis associated 17
Spata31d1d: Spermatogenesis associated 31 subfamily D, member 1D
Speer4e: Spermatogenesis associated glutamate (E)-rich protein 4e
Sry: Sex determining region of Chr Y
Tmco5: Transmembrane and coiled-coil domains 5
Tnp1: Transition protein 1
